# Exploring Care Providers’ Perceptions and Current Use of Telehealth Technology at Work, in Daily Life, and in Education: Qualitative and Quantitative Study

**DOI:** 10.2196/13350

**Published:** 2019-04-22

**Authors:** Hyeyoung Hah, Deana Goldin

**Affiliations:** 1 Department of Information Systems and Business Analytics Florida International University Miami, FL United States; 2 Nicole Wertheim College of Nursing & Health Sciences Florida International University Miami, FL United States

**Keywords:** telehealth technology, nurse practitioners, daily technology use, telehealth care performance, nursing education

## Abstract

**Background:**

A telehealth technology education curriculum designed to integrate information technology and telecommunication well has great potential to prepare care providers for health care delivery across space, time, and social and cultural barriers. It is important to assess the readiness level of care providers to use and maximize the benefits of telehealth technology in the health care delivery process. Therefore, this study explored care providers’ existing experience using technology in various use contexts and compared their familiarity with telehealth technology’s relevant features.

**Objective:**

This study’s objective was to explore care providers’ familiarity with using technology in different settings and their perceptions of telehealth-driven care performance to lay a foundation for the design of an effective telehealth education program.

**Methods:**

The study used quantitative and qualitative analyses. The online survey included four items that measured care providers’ perceptions of care performance when using telehealth technology. Advanced practice registered nurse students rated each item on a 7-point Likert scale, ranging from 1 (“strongly disagree”) to 7 (“strongly agree”). They also responded to three open-ended questions about what kinds of health information technology they use at work, after work, and in their current educational program.

**Results:**

A total of 109 advanced practice registered nurse students responded to the online survey and open-ended questionnaire. Most indicated that using telehealth technology enhances care performance (mean 5.67, median 6.0, SD 1.36), helps make their care tasks more effective (mean 5.73, median 6.0, SD 1.30), improves the quality of performing care tasks (mean 5.71, median 6.0, SD 1.30), and decreases error in communicating and sharing information with others (mean 5.35, median 6.0, SD 1.53). In addition, our qualitative analyses revealed that the students used the electronic health records technology primarily at work, combined with clinical decision support tools for medication and treatment management. Outside work, they primarily used video-text communication tools and were exposed to some telehealth technology in their education setting. Further, they believe that use of nonhealth technology helps them use health information technology to access health information, confirm their diagnoses, and ensure patient safety.

**Conclusions:**

This research highlights the importance of identifying care providers’ existing experience of using technology to better design a telehealth technology education program. By focusing explicitly on the characteristics of care providers’ existing technology use in work, nonwork, and educational settings, we found a potential consistency between practice and education programs in care providers’ requirements for technology use, as well as areas of focus to complement their frequent use of nonhealth technologies that resemble telehealth technology. Health policymakers and practitioners need to provide compatible telehealth education programs tailored to the level of care providers’ technological familiarity in both their work and nonwork environments.

## Introduction

Digital health is known widely for its potential to bring modern health care to the nontraditional, virtual care horizon using mobile health technologies. As one among the medical technology frontiers, telehealth technology “…provides access to health assessment, diagnosis, intervention, consultation, supervision and information across” [1] by allowing users to involve diverse interactions among patients, providers, and specialists over a virtual care platform. Ranging from telephones, facsimile machines, and electronic mail systems to remote patient monitoring devices, users on each end of telehealth technology need to exchange various forms of data and communicate on the mobile platform for virtual care processes. Accordingly, these virtual services require skilled care providers who can manage and analyze multimedia data, make real-time decisions via video or audio communication, and relay such information to other related care provider(s) or team(s).

As telehealth technology is changing modalities of care delivery rapidly, there is a growing expectation that care providers’ use of telehealth technology can play a pivotal role in realizing its benefits and improving the quality of care. As the health care sector is embracing a variety of health technologies and reaching its maturity (eg, electronic health records), more than 70% of health care providers have adopted telehealth technology for their inpatient and ambulatory patient care services [2]. In fact, these providers often have to use new telehealth technology in addition to other technological priorities and responsibilities in care environments, which can be challenging and disruptive. Given how quickly telehealth care services are developing with multiple care modalities [3], care providers need to understand two components of telehealth technology: exchanging electronic information from one site to another [4] and using a wide variety of modalities in telecommunications technologies. Although existing information technology infrastructure facilitates electronic information exchange within health care organizations [5], telehealth telecommunications are more complex, featuring *live conferencing* for interactive two-way communication, *store-and-forward* systems to exchange health information recorded, and *hybrid* mechanisms that feature both live and recorded information care modes [6]. Therefore, it is imperative that care providers are familiar with new technological dynamics, so that they can diagnose and consult with patients by using new telehealth-driven care modalities [7,8].

Under these circumstances, a clinical educational program can nurture care providers’ familiarity with telehealth technology. Advanced practice registered nurses (APRNs) are one of the largest groups of health care providers at the forefront, as their responsibilities involve direct patient care in rapidly changing health care systems. As telehealth technology-driven care services shift the way care is delivered and affect online health care communication between providers and patients and among providers directly, it is critical that future health care providers are educated with well-designed, technologically advanced curriculums [9]. Further, clinical educators should consider the effect technology can have on students’ performance within and beyond the educational settings. Similarly, health profession students should take an active role by expanding their knowledge of health care technology to increase their awareness of its influence in the process of patient care.

Prior studies of provider technology education have focused on incorporating telehealth technology within the curriculum. For example, various factors facilitate and provide barriers to the adoption of telehealth technology [8]. As an education tool, mobile telehealth technology has been shown to help enhance medical students’ virtual communication skills and raise awareness of patient data protection [10]. A recent systematic review showed that although using information and communication technology can help health professionals share clinical practice standards, their perceptions of and behaviors toward the technology vary by the types of technologies they use in different settings [11] and among different care provider groups such as nurse practitioners [12,13]. Given that care providers are using a variety of technologies outside educational settings, it might be possible that their existing experience with similar functions and characteristics of technologies may provide information necessary to design an effective telehealth technology education curriculum, all of which calls for more attention on the subject.

Taken together, we consider the assessment of telehealth technology’s characteristics as a starting point. Telehealth technology resembles other health information technologies, in practice, and daily nonhealth-related technologies. For example, during a live conference with patients, care providers need to diagnose them over a computer screen while scanning the patients’ electronic health information, staring at a computer camera, and answering patients’ text queries. Because nontraditional telehealth care services require providers to use this technology skillfully while seeing and treating patients online, the extent to which they are familiar with similar features of telehealth technology may predict their proper use of telehealth technology in virtual patient encounters. Thus, we examined two important components of a telehealth technology curriculum—experience of using both “focal” and “related” technologies across life-work-education settings—and proposed ways to incorporate this information in designing a tailored telehealth curriculum.

## Methods

### Recruitment

To explore care providers’ level of familiarity with telehealth technology, APRN students in a Graduate Nursing Program at a Southeastern US university were recruited as a study sample. All students in this study are registered nurses pursuing advanced degrees in Nursing Practice, who were recruited because they deliver health care throughout the community currently in various practice settings and using various technological modalities. Specifically, these APRN students have been exposed to telehealth technology in their current education setting. After a Nursing Program leader agreed to participate in the study, a preliminary interview was conducted to contextualize the survey. We interviewed three nursing faculty members and refined the survey questions about technology use in three major domains—daily life, work, and the education program. Final consensus was reached on the number of open-ended questions and refinement of survey instruments. In the process, internal review board approval was obtained to contact and survey the graduate nursing students. As one of the coauthors had a conflict of interests due to her position as a nursing faculty member, the nonnursing author alone was involved in data collection and analyses. Two cohorts of APRN students were then invited to participate in a voluntary online survey during the Spring and Summer semesters in 2018. One cohort started the program in the Spring (primarily in the family program), while the summer cohort was in their final semester of the program across disciplines such as Family Medicine, Psychiatric/Mental Health, Pediatric Medicine, and Adult Geriatric Medicine.

### Survey Instruments

To assess the survey instruments’ face validity, researchers, nurse practitioners, and technologists evaluated and refined our items before pretesting. During the pretest, we collected 20 responses and revised our items for the final survey accordingly. Table 1 presents the questions that the participants responded to using a 7-point Likert scale (1: strongly disagree to 7: strongly agree).

The survey also included open-ended questions that explored individual APRN students’ use of health and nonhealth information technology at work, in their education programs, and for personal use. Participants were asked to name the types of health information technologies that they have used frequently in the three different settings (eg, “Which health information technology do you use in your work setting?”).

### Statistical Analysis

The study generated both quantitative and qualitative results. We sent an online, cross-sectional survey to the two cohorts enrolled in the Graduate Nursing program (a total of 155 students) during the Spring and Summer semesters and had been exposed to telehealth technology in a simulation laboratory. A total of 109 responses were collected from the Spring cohort (n=71) and Summer cohort (n=38), with a 70.32% response rate. All the data were self-reported, so the researchers carefully evaluated nonresponse bias [14,15] and the common method bias [16]. Nonresponse bias occurs when there is a systematic difference between respondents and nonrespondents. We checked nonresponse bias by comparing the difference between early and late responses for each cohort in the Spring and Summer semesters and confirmed that there was no mean difference between them. Second, as we collected independent variable and dependent variable data in the same survey, we further performed the Harman single-factor analysis to assess common method bias. The results showed that one factor explained 35.24% of the variance, confirming no threat of the common method bias. However, not all students answered the demographic or key research questions, resulting in smaller sample sizes.

**Table 1:** Questions used to survey advanced practice registered nursing students’ perceptions of care performance using telehealth technology.

Question #	Survey question
1	I believe that telehealth technology can increase my overall performance on care tasks.
2	I believe that telehealth technology can increase my effectiveness with care tasks.
3	I believe that telehealth technology can increase the quality of dealing with care tasks.
4	I believe that telehealth technology systems can decrease error rates in communicating and sharing information with others.

## Results

### User Statistics

A total of 86 students responded to the demographic questions. As shown in Table 2, 70.9% of these students were female and most (67.4%) were between the ages of 26 and 40 years. Approximately half of the students (49%) were working full-time at a health care organization at the time of the survey, and 32.6% had obtained a master’s degree already.

### Inclusion of Telehealth Technology Use in the Curriculum

#### Exploring Care Providers’ Experience of Using Telehealth-Related Technologies

First, we explored the possibility of including telehealth technology use in the curriculum by looking at care providers’ perceptions of telehealth technology overall. Specifically, we asked whether APRN students believe that using telehealth technology can enhance their care performance at work. Because all respondents had experience with a telehealth technology (ie, doxy.me) in the school’s simulation laboratory, this question was used to ask the students about their existing experience of using telehealth technology and their perceptions of care performance using the technology in the future. Of the 109 students recruited, 100 responded to these questions, and our descriptive statistics showed that most respondents perceive using telehealth technology positively (Table 3). More specifically, APRN students thought that telehealth technology could improve their care performance overall (Question 1: mean 5.67, median 6.0, SD 1.36). In addition, our respondents perceived that technology helped make their care tasks more effective (Question 2: mean 5.73, median 6.0, SD 1.30) and improved the quality of performing care tasks (Question 3: mean 5.71, median 6.0, SD 1.30). Lastly, they also believed that using telehealth at work decreased errors in communicating and sharing information with others (Question 4: mean 5.35, median 6.0, SD 1.53).

**Table 2:** Descriptive statistics of survey respondents.

Variable		n (%)
**Gender**		
	Male	25 (29.1)
	Female	61 (70.9)
**Age (years)**		
	18-25	10 (11.6)
	26-40	58 (67.4)
	41-55	17 (19.8)
	56-65	1 (1.2)
**Education**		
	Bachelor’s degree	51 (59.3)
	Master’s degree	28 (32.6)
	Doctoral degree	2 (2.3)
	Others	5 (5.8)
**Income status (US$ per annum)**		
	25,000-49,999	19 (22.1)
	50,000-74,999	34 (39.5)
	75,000-99,999	8 (9.3)
	≥100,000	9 (10.5)
	Prefer not to answer	16 (18.6)
**Occupational status**		
	Working full-time	49 (57.0)
	Working part-time	31 (36.0)
	Unemployed	4 (4.7)
	Unable to work	1 (1.2)
	Other	1 (1.2)

#### Existing Experience of Using Technology via Qualitative Textual Analysis

Next, we delved further into the types of technologies that care providers have used across multiple domains to identify their similarities and differences. In our survey, each respondent was asked to list the names of technology they have used at work, in daily life, and in educational contexts (Tables 4-6).

According to the 78 respondents who answered this question completely (Table 4), the technology they used most frequently in their work was electronic health record systems from different vendors (24.36%) such as Epic, Cerner, and eClinical Works. Furthermore, they used evidence-based clinical decision support tools: 17% reported using Epocrates and 14% used Wikipedia-type reference tools such as UpToDate. Interestingly, 2% of respondents used a telehealth app (eg, Care on Demand). Other categories included diverse responses such as Sanford guide and health4 me.

During their educational program, respondents seemingly used a similar variety of health information technologies from their work place (Table 5).

In our last open-ended question, APRN students were asked to name nonhealth care technologies that they use in their daily lives, such as the internet, Facebook, Twitter, YouTube, and text messages. As respondents did not specify the names of apps that they use after work hours, we reported their frequency of using technology across broad categories of nonhealth technologies. As shown in Table 6, 88 APRN students used social network and chatting apps most frequently (72% for daily use), followed by internet banking (43% for weekly use) and transportation apps (44% for monthly use).

**Table 3:** Descriptive statistics of survey questions and responses.

Questions and responses		n
**Question 1**		
	Strongly disagree	4
	Disagree	1
	Somewhat disagree	1
	Neither agree nor disagree	5
	Somewhat agree	22
	Agree	41
	Strongly agree	26
**Question 2**		
	Strongly disagree	4
	Disagree	2
	Somewhat disagree	2
	Neither agree nor disagree	22
	Somewhat agree	45
	Agree	25
**Question 3**		
	Strongly disagree	3
	Disagree	1
	Somewhat disagree	2
	Neither agree nor disagree	4
	Somewhat agree	23
	Agree	40
	Strongly agree	27
**Question 4**		
	Strongly disagree	3
	Disagree	3
	Somewhat disagree	7
	Neither agree nor disagree	10
	Somewhat agree	22
	Agree	30
	Strongly agree	25

**Table 4:** Summary of health technology types used in the workplace.

Technology	Tool	n (%)
Electronic Health Records	Epic, Cerner, Allscripts, mychart	19 (24.35)
Epocrates	Clinical Decision Support	13 (16.67)
Medscape	Clinical Portal	4 (5.12)
UpToDate	Drug Reference	11 (14.10)
CDC^a^ Vaccine Schedules	Vaccine Reference	4 (5.12)
Care on Demand	Telehealth App	2 (2.56)
Other	Sanford guide, health 4 me, etc	25 (32.05)

^a^CDC: Centers for Diseases Control and Prevention.

**Table 5:** Summary of the health technology types used in the education program.

Technology	Tool	n (%)
Electronic Health Records	Epic, Cerner, etc	14 (25.00)
Epocrates	Clinical Decision Support	8 (14.29)
Uptodate	Drug Reference	3 (5.36)
School websites	None	7 (12.50)
Simulation laboratory technology	School Technology including Telehealth Technology	4 (7.14)
Typhon	Student Tracking System	6 (10.71)
Others	Familydoctor.org, PharmaxSoft, np notes, etc	14 (25.00)

**Table 6:** Summary of health technology types used in daily life.

Technology category	Daily, n (%)	Weekly, n (%)	Monthly, n (%)	Never, n (%)
Internet banking	39 (44.32)	38 (43.18)	10 (11.36)	1 (1.14)
Online shopping	21 (23.86)	34 (38.64)	28 (31.82)	5 (5.68)
Social network	63 (71.59)	16 (18.18)	5 (5.68)	4 (4.55)
Entertainment	40 (45.45)	26 (29.55)	11(12.50)	11 (12.50)
Chatting	63 (71.59)	8(9.09)	7 (7.95)	10 (11.36)
Transportation	14 (15.91)	19 (21.59)	39 (44.32)	16 (18.18)

Lastly, to explore the students’ perceptions of the effect of using nonhealth technology daily on the use of health technology at work, we sent the students a follow-up question in an email survey, in which each respondent was asked, “When you recall using apps such as banking apps, social media apps, entertainment apps, communication apps, transportation apps, or chatting apps in your daily lives, do you believe such non-health technology use influences health information technology (HIT) use at your current work place? If the answer is yes, how specifically does using apps in your personal life influence your patient care performance at work?” Of the 109 students, 14 answered this question. Of them, 3 responded that using apps outside the classroom helped them feel comfortable and more confident in caring for patients:

I would learn more through using apps to help my patients.

It helps with everything.

I am young and using apps is comfortable to me.

To the second follow-up question, 9 students answered that the use of apps in multiple domains enables them to access health information, confirm diagnoses and tests, and maintain patient care safety easily (Textbox 1).

Taken together, our quantitative and qualitative results indicated that (1) APRN students believe the use of telehealth technology can increase their care performance, (2) they are familiar with features of recording/managing patient information via EMR as well as decision-support tools to identify medical symptoms and treatment for clinical decision making at work, (3) they use similar technologies in their education setting, and (4) most students use social network and chatting apps on a daily basis (72% for both categories) in daily life and believe that using apps in daily life makes them feel confident about accessing health information by using the workplace health technology at the time of care. In the next section, we discuss how a new technology curriculum can integrate this information.

**Quotes from the responses of 9 students to the second follow-up question.**

“It advantages since the information is accessible.”

“More access than my head can hold.”

“Helps to confirm your recall especially a test and confirm what you are doing.”

“To check pharm is for safer for patients…”

“My comfort level makes it easier for me to use apps.”

“I am comfortable to search for things at work.”

“Provide safer care…”

“Confirm diagnosis and tests…”

“I can be more accurate with medications and check for safety interactions.”

“My head cannot hold all the information I need so it helps me to help the patient.”

“To look for information to diagnose and look for medications, to do everything.”

## Discussion

### Principal Results

As the first step in designing a technology education curriculum, this research explored care providers’ familiarity with telehealth technology by considering their level of exposure to using various features of both health- and nonhealth-related technologies in different settings. Acknowledging the scarcity of research in this domain, our study focused particularly on the characteristics of telehealth technology, which combines various technological features such as electronic health data management, real-time communication using multimedia, and chatting without hybrid formats, and further traced the system types that care providers have used across different settings. The results from both the quantitative and qualitative analyses showed that care providers have positive attitudes about telehealth technology’s ability to enhance their care performance with respect to overall task performance, effectiveness of care, quality of care tasks, and sharing information. Further, technologies for personal use shared similarities with the focal telehealth technology, nurturing their abilities to communicate virtually and manage multimedia images, as well as highlighted the benefits of using nonhealth technology in life when using telehealth technology at work.

**Figure 1:**
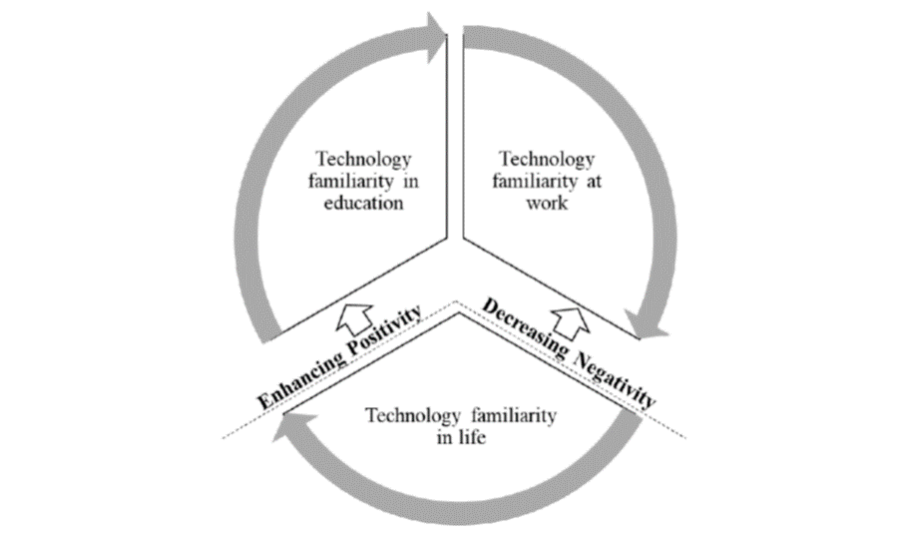
Incorporating technology familiarity at work, in life, and in education programs.

Although the study was exploratory, our explicit focus on care providers’ use of technology in the three domains suggests that a telehealth education curriculum can be complemented by the use of other similar technology. In summary, our findings showed that (Figure 1): (1) there is strong overlap in technology familiarity between workplace and education programs and (2) daily experience of using nonhealth technology does not mesh with the existing nursing education, although care providers use mobile technology features daily that share commonalities with telehealth technology features. Thus, we propose that a telehealth curriculum needs to incorporate the existing experience of using technology, promoting similarity between telehealth technology and daily technology use. More specifically, adding components of social media and virtual communication to the existing nursing education may enhance the familiarity with a telehealth technology. For example, including social media channels to promote communication among students and between faculty members and students may promote their positive experiences with the similar features of telehealth technology [17]. As another example, to alleviate any negative perceptions such as frustration with technology about using technology overall, adding YouTube-based instructions on trouble shooting for telehealth can help enhance care providers’ autonomy in processing necessary information about a new technology [18].

From a practical standpoint, it is timely to assess care providers’ technology experience about their increasing role at the forefront of using virtual patient care modalities, particularly with rural or underserved populations [12]. It is important to understand APRN students’ use of technology in the workplace, daily life, and educational programs to gain an understanding of their ability to adapt to the health care arena, as this arena will require them to use and be increasingly adaptable to technology in order to facilitate patient communication and improve patient safety and practice outcomes. Many health technologies have transformed professional health care practices, which alters the educational requirements for future practitioners. A flexible education program employing new technologies in the market in a timely manner has helped care providers familiarize themselves with various features from such health technologies and increase their comfort level [19-21]. For example, many educators use personal digital assistants in clinical education to take advantage of the current technology to enhance care providers’ learning outcomes [22-24]. More recently, a video-based education program was shown to increase nursing students’ satisfaction and learning experiences [25]. Furthermore, the telehealth curriculum has suggested the inclusion of problem-based solving and telehealth site visits in the curriculum [26].

However, development of a new technology program is not an easy task. Considering the fast-moving technology advances in the health care arena [27], a technology curriculum for health profession students may not keep up with the speed and variety of the prevalent health technologies. Moreover, care providers’ technological ability to combine technology use and health care services is still neither defined well nor instructed frequently. An alternative educational focus on “related” technology experience in daily life may allow health care educators to take a proactive approach to incorporating newly developed health technology into education settings. In particular, health care educators can identify similar, widely available technologies that provide similar technology experience in the education setting and encourage providers to use them in training, all of which can help care providers enter the workforce with positive attitudes about new workplace technology.

Health care information technology vendors should also consider care providers’ familiarity and experience with health as well as nonhealth information technologies for the design of a new health technology in the market. This is closely related to system usability, and a well-designed telehealth technology providing additional care monitoring and decision support capabilities can reduce care providers’ frustration toward the technology [28]. Addition of familiar features may encourage care providers to reduce the level of frustration about health technology and adopt a new health technology at work.

### Limitations

This research was an initial pilot study for a grant proposal. First, given the time and resources available when the study was conducted, only APRN students (n=109) in limited specialties such as Family Medicine participated. Future researchers should consider recruiting more APRN students with diverse specialties. Second, to capture care providers’ continuous use of various domain technologies, their technology use behaviors should be tracked over time and analyzed using a cross-sectional time-series analysis. Third, a more in-depth survey is necessary to capture providers’ daily use of nonhealth technology to compare technology use in both work and nonwork contexts. As participants self-tracked their use of various technologies across three different settings based purely on their memory and willingness, more robust data collection will enhance this study’s qualitative analysis findings. Finally, in this paper, we identified the importance of considering care providers’ technology experience across contexts and their care performance using telehealth technology. Future research may examine whether and how experience can influence attitudes and behaviors related to using telehealth technology through robust statistical analysis.

### Conclusions

This study provided insights to inform a new telehealth nursing education program by exploring nursing students’ technology use characteristics at work, in daily life, and in an educational setting. These findings contribute to the health education literature and to health policy initiatives by demonstrating a new approach to incorporate care providers’ existing experience of using cross-domain technologies to design a tailored telehealth technology curriculum.

## Abbreviations

APRN: advanced practice registered nurses
CDC: Centers for Diseases Control and Prevention
